# Antioxidant Activity of Vitamin C against LPS-Induced Septic Cardiomyopathy by Down-Regulation of Oxidative Stress and Inflammation

**DOI:** 10.3390/cimb44050163

**Published:** 2022-05-23

**Authors:** Ayed A. Shati, Mohamed Samir A. Zaki, Youssef A. Alqahtani, Saleh M. Al-Qahtani, Mohamed A. Haidara, Amal F. Dawood, Asmaa M. AlMohanna, Mahmoud H. El-Bidawy, Muhammad Alaa Eldeen, Refaat A. Eid

**Affiliations:** 1 Department of Child Health, College of Medicine, King Khalid University, Abha P.O. Box 62529, Saudi Arabia; ashati@kku.edu.sa (A.A.S.); yal-qahtani@kku.edu.sa (Y.A.A.); smuadi@kku.edu.sa (S.M.A.-Q.); 2 Anatomy Department, College of Medicine, King Khalid University, Abha P.O. Box 62529, Saudi Arabia; mszaki@kku.edu.sa; 3 Department of Histology and Cell Biology, College of Medicine, Zagazig University, Zagazig 31527, Egypt; 4 Department of Physiology, Kasr Al-Aini College of Medicine, Cairo University, Cairo 11519, Egypt; haidaram@cu.edu.eg (M.A.H.); elbidawy@psau.edu.sa (M.H.E.-B.); 5 Department of Basic Medical Sciences, College of Medicine, Princess Nourah Bint Abdulrahman University, Riyadh P.O. Box 84428, Saudi Arabia; afdawood@pnu.edu.sa (A.F.D.); amalmohanna@pnu.edu.sa (A.M.A.); 6 Department of BMS, Division of Physiology, College of Medicine, Prince Sattam Bin Abdulaziz University, Al-Kharj P.O. Box 11942, Saudi Arabia; 7 Cell Biology, Histology & Genetics Division, Zoology Department, College of Science, Zagazig University, Zagazig 44519, Egypt; muhammadalaaeldeen@zu.edu.eg; 8 Pathology Department, College of Medicine, King Khalid University, Abha P.O. Box 62529, Saudi Arabia

**Keywords:** cardiac tissue, vitamin C, histopathology, oxidative markers, electron microscopy, statistical analysis

## Abstract

In severe cases of sepsis, endotoxin-induced cardiomyopathy can cause major damage to the heart. This study was designed to see if Vitamin C (Vit C) could prevent lipopolysaccharide-induced heart damage. Eighteen Sprague Dawley male rats (*n* = 6) were divided into three groups. Rats received 0.5 mL saline by oral gavage in addition to a standard diet (Control group), rats received one dose of endotoxin on day 15 (lipopolysaccharide) (LPS) (6 mg/kg), which produced endotoxemia (Endotoxin group), and rats that received 500 mg/Kg BW of Vit C by oral gavage for 15 days before LPS administration (Endotoxin plus Vit C group). In all groups, blood and tissue samples were collected on day 15, six hours after LPS administration, for histopathological and biochemical analysis. The LPS injection lowered superoxide dismutase (SOD) levels and increased malondialdehyde in tissues compared with a control group. Furthermore, the endotoxin group showed elevated inflammatory biomarkers, tumor necrosis factor-α (TNF-α) and interleukin-6 (IL-6). Both light and electron microscopy showed that the endotoxic-treated group’s cardiomyocytes, intercalated disks, mitochondria, and endothelial cells were damaged. In endotoxemic rats, Vit C pretreatment significantly reduced MDA levels and restored SOD activity, minimized biomarkers of inflammation, and mitigated cardiomyocyte damage. In conclusion: Vit C protects against endotoxin-induced cardiomyopathy by inhibiting oxidative stress cytokines.

## 1. Introduction

Sepsis is a potentially fatal infection-related disorder malfunctioning host response [1]. At least 19 million individuals are threatened with sepsis worldwide, reminding scientists and doctors that it remains a serious global health concern and therapeutic dilemma [2,3]. Regardless of extensive research into advances in intensive care and supportive technologies, sepsis is still among the causes of morbidity and mortality in non-coronary intensive care units for critically ill patients [4,5]. A dysregulated inflammatory response, oxidative stress, calcium regulatory disorder, dysregulated autonomic nervous system, defective autophagy, apoptotic damages, and mitochondrial and endothelial dysfunction are all symptoms of sepsis-induced cardiomyopathy [6,7,8].

Endotoxins are complexes made up of LPS that are the principal component of Gram-negative bacteria’s outer wall and are actively secreted by bacteria [9,10]. By stimulating inflammatory mediators, endotoxin can cause sepsis leading to organ damage over time [11,12]. 

One of the most well-known organ dysfunctions in sepsis is cardiac dysfunction. Although the mechanism of the myocardial malfunction is complex and poorly understood, accumulating experimental data suggest that diminished ventricular myofibril reactivity, NO-peroxynitrite activation, and the inhibition of mitochondrial oxidative phosphorylation are the fundamental intracellular mechanisms [13]. The familiar technique by which the septic response damages the tissues is believed to be frequent vascular endothelial injury and microthrombosis. As a result, the tissues receive less oxygen and substrate, leading to the generation of free oxygen radicals and anaerobic metabolism [14]. Reduced membrane fluidity and function, impaired mitochondrial and Golgi apparatus activities and enzyme inhibition are toxicological outcomes of lipid peroxidation [15]. 

Vit C is a supplement with both radical scavenging and antioxidant properties [16]. Therapy significantly enhanced the ejection fraction of the left ventricle in patients with heart failure [17] and a cohort of Spanish graduates; there was a reduction in cardiovascular mortality [18]. Vit C’s antioxidant properties help to prevent and treat cardiovascular diseases [19]. Antioxidant systems include antioxidant vitamins A, C, and E, glutathione (GSH), glutathione peroxidase (GSH-Px), ceruloplasmin, superoxide dismutase (SOD) and catalase (CAT), safeguard cells from lipid peroxidation, which is at the basis of a variety of pathological diseases [20]. Vitamins are appropriate antioxidants for improving tissue protection against oxidative stress due to their easy, efficient, and safe dietary supplementation in a broad range of dosages without potential complications [21].

TNF-α and IL-6 increased plasma levels and were linked to diastolic dysfunction in the left ventricle. The pathogenesis of diastolic dysfunction may be influenced by an active proinflammatory process [22]. TNF-α and IL-6 interactions reduce eNOS phosphorylation and boost oxidative stress, resulting in coronary endothelium dysfunction [23].

The objective of this research was to evaluate if vitamin C could protect rats from oxidative cardiac tissue damage throughout experimentally induced endotoxemia.

## 2. Materials and Methods

### 2.1. Test Substance

Endotoxin (lipopolysaccharide from Salmonella abortus equi) was supplied by Sigma Chemical, St. Louis, MO, USA. Louis, MO, USA, supplemented the Vit C.

### 2.2. Animals

The rats used in this investigation were 18 Sprague Dawley male rats weighing 150–250 g. Standard diet and water were given on an ad libitum basis. The animals were divided randomly into three groups (*n* = 6). The control group rats received only a standard diet and 0.5 mL saline by oral gavage for 15 days. Single dose of LPS (6 mg/kg BW) was administered to the endotoxin group intraperitoneally on day 15, and the endotoxin plus Vit C group received Vit C 500 mg/kg BW/day [24] solubilized in saline via oral gavage and delivered intraperitoneally for 15 days, followed by a single dose of LPS on day 15. Six hours following LPS injection, blood samples were gathered from the retro-orbital vein and tissues from the heart were extracted under phenobarbitone anesthesia for biochemical and histopathological examination [25].

### 2.3. Oxidative Stress (MDA and SOD) & Inflammatory Biomarkers (TNF-α and IL-6) Assessment

To measure oxidative stress and inflammatory biomarkers, cardiac specimens were homogenized in ice-cold saline and centrifuged for 15 min at 18,000× *g* (148C). The TBARS Assay Kit was used to measure MDA (Item No. 10009055, Cayman Chemical Company, Ann. Arbor, MI, USA). The kit was used to measure SOD (Item No. 706002, Cayman Chemical Company, Ann. Arbor, MI, USA). According to the manufacturer’s instructions, TNF-α was tested by applying an ELISA kit (BIOTANG INC. Cat. No. R6365, St, Lexington, MA, USA). IL-6 was quantified using an ELISA kit (BIOTANG INC, Cat. No. RB1829, St, Lexington, MA, USA).

### 2.4. Hematoxylin–Eosin Staining of the Heart Cross-Sections

Heart tissue samples were harvested and preserved in neutral buffered formalin (10 per cent) for 24 h. After dehydrating tissues with increasing alcohol concentrations, paraffin blocks were created. For histological investigation, tissue sections (5 µm thick) were stained with hematoxylin and eosin [26].

### 2.5. Transmission Electron Microscope (TEM) 

Small pieces 1 mm^3^ of the heart were preserved and fixed at 4 °C in a 2.5 per cent glutaraldehyde solution in 0.1 M sodium cacodylate buffer, pH 7.2. For two hours, the samples were postfixed in a 1% osmium tetroxide in 0.1 M sodium cacodylate buffer, pH 7.2 at 4 °C, before dehydration in ascending grades of ethyl alcohol before initiating the embedding in Spur’s resin. The tissue blocks were dissected into 80 nm thick ultrathin sections. The samples were stained with uranyl acetate and lead citrate, then analyzed under an electron microscope (JEM-1011, Jeol, Tokyo, Japan) operating at 80 Kv [27].

### 2.6. Statistical Analysis

The mean and standard deviation (SD) were used to represent the data. Graph Pad Prism program (Version 6) was used to analyze the data. Tukey’s post hoc test was processed after performing a one-way ANOVA. To implement this, a possible relevance between two separate parameters, a statistical examination of Pearson correlation was carried out. If *p* ≤ 0.05, the results were considered significant.

## 3. Results

### 3.1. Biochemical Results

**Figure 1:**
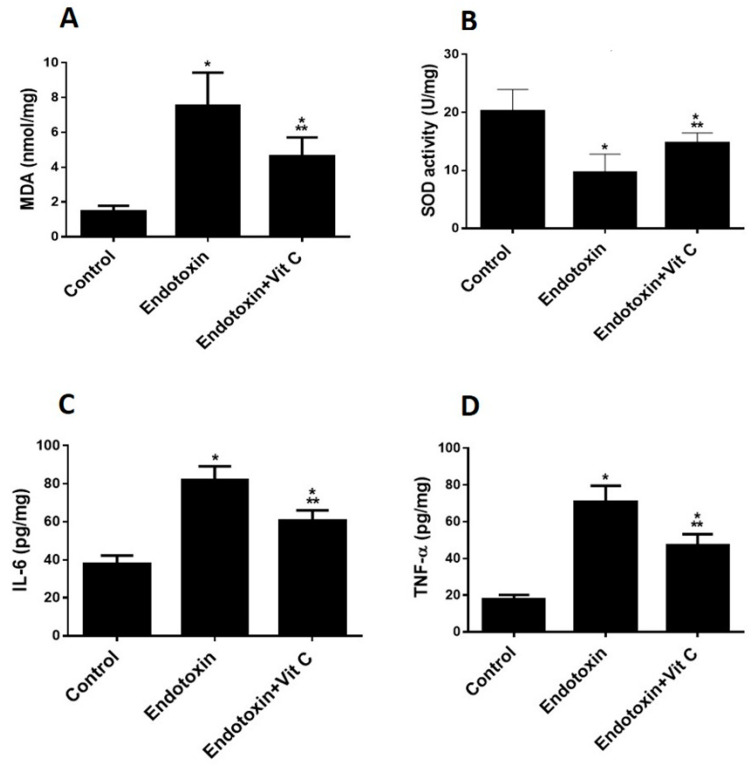
**Biochemical parameters (Oxidative stress and proinflammatory biomarkers) of all groups.** (**A**,**B**): Vit C protects against endotoxin-induced oxidative stress and decreases biomarkers of oxidative stress (MDA (**A**) and SOD (**B**)) in heart homogenates of the three studied groups. The findings are the mean (±SD), *n* = 6. * *p* < 0.05 when compared to the controls, *** *p* < 0.05 in contrast to endotoxin group. (**C**,**D**): Vit C protects against endotoxin-induced inflammation and decreases the pro-inflammatory biomarkers (IL-6 (**C**) TNF-α (**D**)) in heart homogenates of the three studded groups. The findings are the mean (±SD), *n* = 6. * *p* < 0.05 when compared to the controls, *** *p* < 0.05 in contrast to endotoxin group.

Increased oxidative stress was shown in the endotoxin group, as can be seen from increased MDA and reduced SOD against the control group (*p* ≤ 0.05) (Figure 1A,B). Vit C supplementation significantly reduced MDA (indicative of peroxidation) and increased the antioxidant enzyme (SOD) compared to the endotoxin group, but not to control levels.

As indicative of inflammation, the group that was exposed to endotoxins demonstrated increased inflammatory biomarkers (TNF-α & IL-6 cytokines) versus the controls (*p* ≤ 0.05) (Figure 1C,D). Supplementing with Vit C contributed to a considerable reduction of proinflammatory biomarkers compared to the endotoxin group but not back to control levels.

### 3.2. Histological Examination

**Figure 2:**
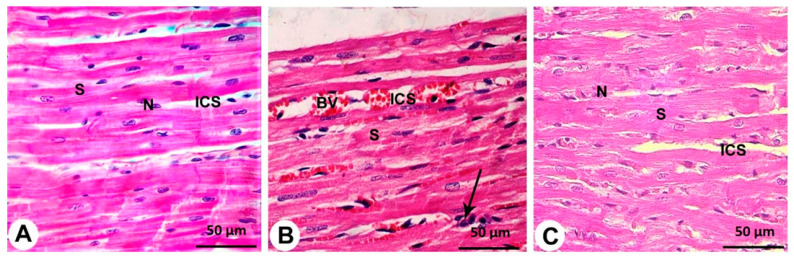
**Histopathological evaluation of cardiac architecture from all groups (400×).** The control group (**A**) shows centrally located nuclei (N), branching cytoplasmic network with striations (S) and delicate connective tissue between the muscle fibers (ICS) with few capillaries (**C**). The endotoxin group (**B**) shows more apparent alterations, where most of the cardiac muscle fiber striations (S) have disorganization and fragmentation, increment of nuclear peripheralization and pyknosis (N) together with sarcoplasmic vacuolation. Increased interstitial space (ICS) with congestion and dilation of the blood capillaries (BV) and monocellular infiltration (arrow) are displayed. Endotoxin plus Vit C treated group (**C**) shows the approximately normal histological appearance of cardiac myocyte striations (S), minimal interstitial space (ICS) and decreased congestion of blood capillaries. Some pyknotic nuclei (N) are still seen.

Cardiomyocytes from the control group were striated and organized in a linear array with acidophilic cytoplasm, a precise branching pattern (sheet-like) and oval nuclei in the center with a thin layer of connective tissue separated them from the well-established cardiac blood capillaries (Figure 2A).

Endotoxin-damaged cardiomyocytes were disordered, degraded, and disorganized, with different degrees of hypertrophy. In most of the myocytes, the nuclei were pyknotic and encircled by perinuclear cytoplasmic vacuolation with pale acidophilic sarcoplasm. Congested, dilated, and expanded blood capillaries were wrapped by interfibers. Cellular infiltration of mononuclear cells in the perivascular space, hemorrhage and interstitial edema were also detected (Figure 2B). 

There were few inflammations, myonecrosis, edema, and red blood cell extravasation in the endotoxin with the Vit C group, indicating that they had the lowest percentage of myocardial damage. Many well-organized muscle fibers with centrally vesicular nuclei were identified in certain areas, but a few had peripheral dark nuclei and slightly rarified cytoplasm. Blood vessels were intact; however, there were some alterations between muscle fibers (Figure 2C). 

### 3.3. Transmission Electron Microscope (TEM) Analysis

**Figure 3:**
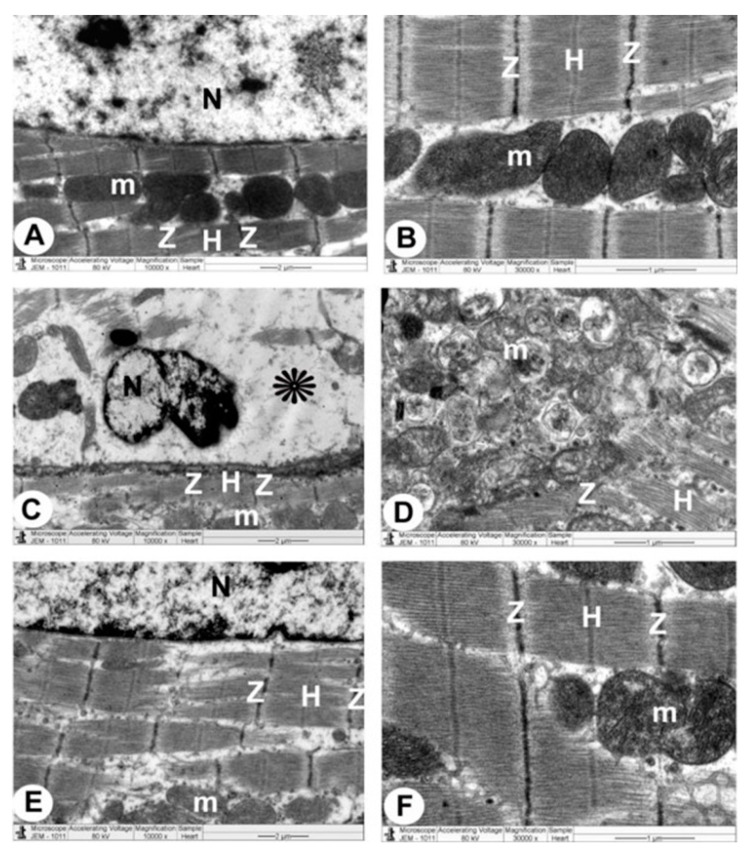
**TEM micrographs of cardiac architecture from all groups.** The control group (**A**,**B**) show a normal myocardial striation architecture with well-preserved mitochondrial (m) integrity. Preserved nucleus (N) with a normally distributed chromatin and nuclear envelope, abundant cytoplasm packed with intact myofibrils and a striated pattern with obvious (Z and H) bands can be noticed. Endotoxin group (**C**,**D**) exhibits swollen mitochondria (m) with disorganized cristae with flocculent density deposition, defects in the outer mitochondrial membrane, and disrupted myofibrils with disarrangement of muscle (Z and H) bands. Disassembly of the nuclear envelope, random dispersion of clumped chromatin in nuclei (N) and marked spaces interstitially (*) interstitially are also observed. Endotoxin plus Vit C group (**E**,**F**) demonstrates a typical architecture with well-preserved integrity and a striated pattern of clear (Z and H) bands, nucleus (N) with proper chromatin organization, and nuclear envelope and sustained mitochondria (m).

**Figure 4:**
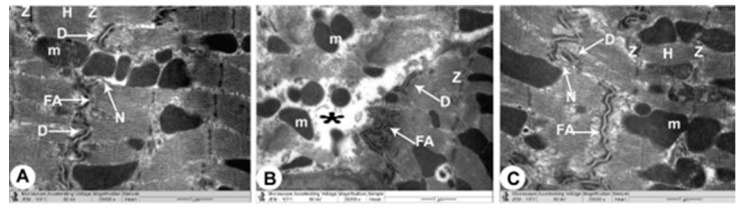
**TEM micrographs of the intercalated disc from all groups.** Control group (**A**) displays a healthy cardiomyocyte with abundant cytoplasm and healthy myofibrils, as well as a striated pattern with clear (Z and H) bands and undamaged mitochondria (m). Intercalated disc with characteristic fascia adherent (FA), desmosomes (D) and Gap (nexus) junctions (N) are also seen. The endotoxin group (**B**) exhibits disorganized myofibril cytoplasm with muscle (Z and H) band damage with pleomorphic mitochondria (m). Damaged intercalated discs with marked disruption of their constituents (fascia adherent (FA), desmosomes (D) and gap junction (Nexus) (N)) are also noticed. Endotoxin plus Vit C group (**C**) shows intact myofibrils, clear (Z and H) bands with a striated pattern, mitochondria (m) and intercalated discs (fascia adherent (FA), desmosomes (D) and Gap (nexus) junctions (N)) are also evaluated.

**Figure 5:**
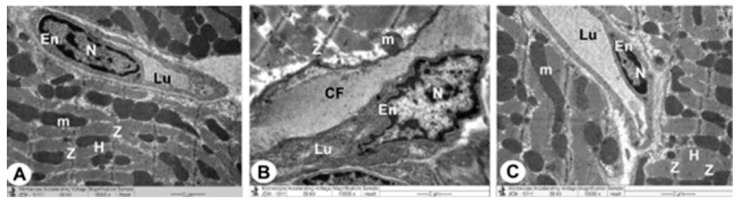
**TEM micrographs of the mitochondria, Z and H band and endothelial cells from all groups.** The control group (**A**) shows a normal myocardial striation architecture with well-preserved mitochondrial (m) integrity. Healthy myofibrils with a striated pattern and distinct bands (Z and H) are found in a packed cytoplasm, and capillaries are composed of simple squamous endothelial cells (En) with their nucleus (N). The endothelial cells are connected by overlapping to form a vascular lumen (Lu). The endotoxin group (**B**) displays enlarged mitochondria (m), more distorted capillaries with deformed endothelial cells (En) and disturbed myofibrils with disarrangement of muscle bands (Z and H) that form vascular lumen (Lu) with its disrupted irregular nucleus (N). Note that deposition of collagen fibrils (CF) is seen. Endotoxin plus Vit C group (**C**) reveals typical architecture with well-preserved integrity and a striated pattern of clear bands (Z and H), preserved mitochondria (m), intact capillaries with normal endothelial cells (En) that form vascular lumen (Lu) and a healthy nucleus (N).

The control group’s ventricular myocytes were branching, striated and connected at the intercalated discs (ICDs). Myofibrils were organized within the sarcomeres in a regular pattern and between the bands (Z and H). Interdigitations, which are the ICDs’ finger-like folds, were repeated at regular intervals. There were fasciae adherents, spot desmosomes and gap junctions (nexus) within the interdigitations. Furthermore, uniformly scattered mitochondria were observed as chain-like structures linked to sarcomere length. Mitochondria were oval organelles with transversally oriented cristae and a relatively electron-dense matrix that formed clusters along the myofibrils in the cardiomyocytes. Ventricular myocyte nuclei were euchromatic, with chromatin that was homogenous, loose and coarsely granular. A dense coating of chromatin masses was also observed on the inner surface of the nuclear membrane. Simple squamous endothelial cells with their regular nuclei consisting of capillaries were clarified. By overlapping and attaching, endothelial cells produced a vascular lumen (Figure 3A,B, Figure 4A and Figure 5A).

**Figure 6:**
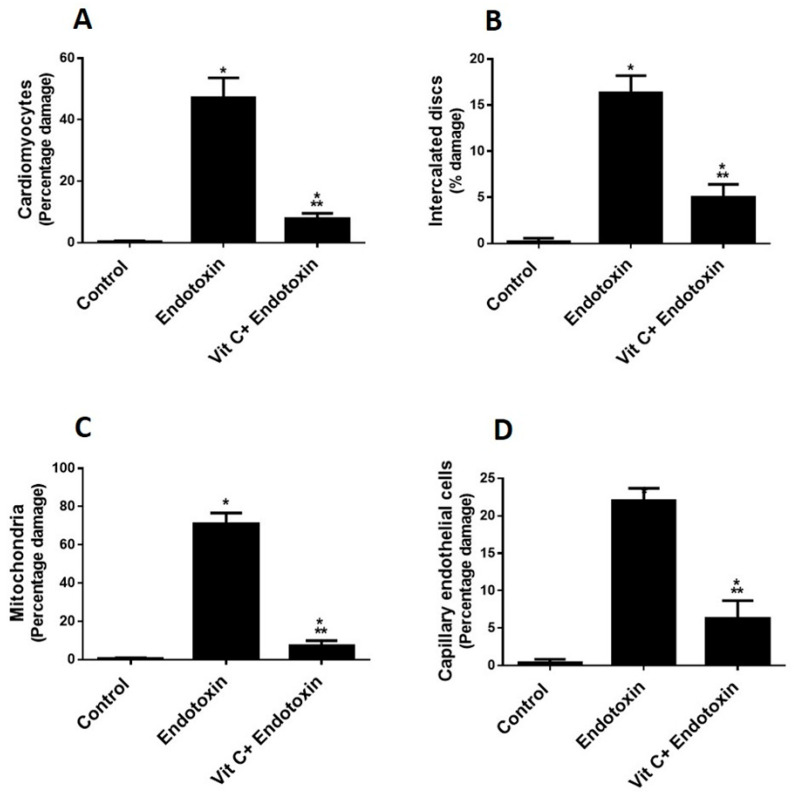
**The percentage of TEM cardiac, mitochondrial, capillary endothelial damage.** Vit C protects against endotoxin-induced cardiomyocytes (**A**), intercalated discs (**B**), mitochondrial (**C**) and capillary endothelial cell (**D**) damage. * *p* < 0.05 when compared to the controls, *** *p* < 0.05 in contrast to endotoxin group.

TEM photomicrographs of the endotoxin groups showed extensive damage in specific ventricular myocardium cells (Figure 6A); and some myofibril thinning and rupture. Furthermore, ICDs (Figure 6B) with fascia adherent, desmosomes and gap junction were distributed over the tissue haphazardly, primarily in the vicinity of the adherent junction, with comparatively maintained desmosomal intercellular communications with degenerative mitochondrial alterations (Figure 3B, Figure 4B and Figure 6C). The inner membrane of the mitochondria expanded in most muscle cells, inducing cristae fragmentation. The nuclear membrane’s disassembly contributed to the creation of clumped heterochromatin that spread randomly throughout the damaged nuclei. Lesser vacuoles and gaps interstitially were seen. The vascular lumen’s endothelial cells were injured, with a fragmented irregular nucleus and collagen fibrils deposited (Figure 3C,D, Figure 4B, Figure 5B and Figure 6D).

The endotoxin plus Vit C group showed improvement as opposed to the endotoxin group and experienced the regeneration of cardiac muscle fibers. The myocyte nuclei appeared oval euchromatic, and the ultrastructure of the ICDs and gap junction was retained with intact interdigitation and fascia adherent. Only some mitochondria appeared to be enlarged with variations in size and shape, which correlated with partially destroyed cristae, while almost all of the mitochondria kept their entire cristae and dense matrix. There were intact capillaries with typical endothelial cells forming the vascular lumen and a healthy nucleus (Figure 3E,F, Figure 4C and Figure 5C). In Figure 6A–D, cardiomyocytes, ICDs, mitochondria, and capillary endothelial cells demonstrated a percentage damage reduction.

### 3.4. The Correlations between Heart Damage, Oxidative Stress and Proinflammatory Biomarkers

**Figure 7:**
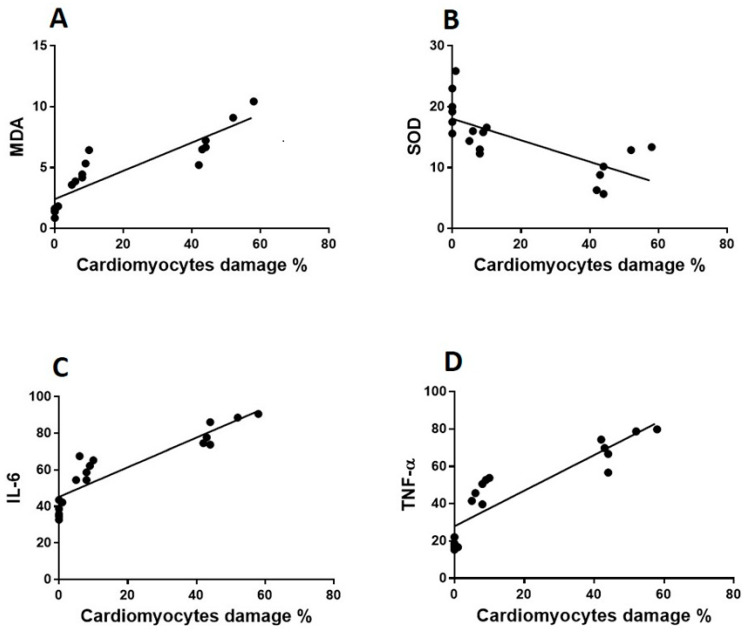
**Correlation between cardiomyocytes and oxidative stress and inflammatory biomarkers.** MDA, SOD, IL-6 and TNF- α levels of cardiac homogenate were estimated in all rat groups. The correlation between the percentage of cardiomyocyte damage and oxidative stress biomarkers (MDA and SOD) are shown in (**A**,**B**), respectively. The correlation between the percentage of cardiomyocyte damage and proinflammatory cytokines (TNF-α and IL-6) have been reported in (**C**,**D**) respectively.

**Figure 8:**
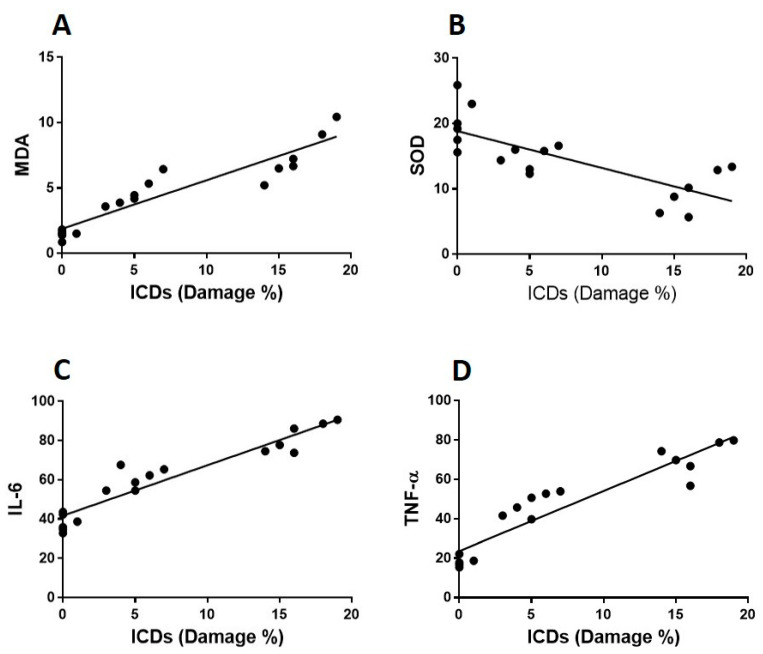
**Correlation between cardiomyocyte intercalated discs (ICDs) and biomarkers of oxidative stress and inflammation.** MDA, SOD, IL-6 and TNF-α heart homogenate levels were calculated in all rat groups. The correlation between the percentage damage of ICDs and oxidative stress biomarkers (MDA and SOD) are shown in (**A**,**B**), respectively. The correlation between the percentage damage of ICDs and proinflammatory cytokines (TNF-α and IL-6) has been demonstrated in (**C**,**D**), respectively.

The authors explored the correlation between cardiomyocyte tissue damage and the concentrations of oxidative and proinflammatory biomarkers in cardiomyocytes to see if there was a correlation between the pathogenesis of LPS-induced tissue injury and these biomarkers and also to determine whether there was a link between Vit C’s protective effect against LPS-induced cardiomyopathy (Figure 7 and Figure 8). Cardiomyocyte scoring identified a negative relationship with SOD (r = 0.7; *p* < 0.008), whereas, MDA (r = 0.8; *p* < 0.001), IL-6 (r = 0.9; *p* < 0.001) and TNF-α (r = 0.8; *p* < 0.001) displayed a positive and significant correlation.

## 4. Discussion

We investigated the effect of Vit C in a rat model to prevent endotoxemia-induced cardiac damage using histological, ultrastructural and oxidant/antioxidant features. Our results revealed that in the endotoxin group, there was an increase in MDA (an indicator of lipid peroxidation) and a decrease in the antioxidant enzymes SOD.

The increase in the generation of reactive oxygen species (ROS) is attributed to oxidative stress (OxS) and a reduction in antioxidant enzymes [28]. A decline in the number or activity of antioxidant enzymes leads to ROS increase, which creates oxidative tissue damage [29]. 

The process by which endotoxin promotes cardiac damage is thought to be due to a reduction in antioxidant enzyme activity in cardiac tissues; in addition, reactive oxygen species (ROS) elevation enhances the synthesis of glycation end-products [30] and triggers cardiac damage [31].

Oxidative stress and elevated levels of proinflammatory cytokines may create considerable damage to the cell membrane [32,33]. A study on this topic supports this, which reveals an increase in Il-6 and TNF-α in the cardiac tissue homogenate of the endotoxin group. This could explain why the endotoxin group’s heart ultrastructure was damaged.

We found that giving Vit C to an endotoxin group safeguarded the rats from increased oxidative stress and inflammatory biomarkers, explaining why this antioxidant helps prevent endotoxemia-induced structural heart changes.

The endotoxemic rats’ heart specimens underwent histological investigation, which demonstrated pathological manifestations in all. Really visible alterations were a remarkable enlargement of the intercellular spaces along and reorganization of the heart architecture which supports prior research that reported enlarged interstitial tissue in heart muscles due to endotoxemia that is related to the existence of more connective tissue elements, particularly collagen fibers and was linked to the severity of muscle injury [32]. Endotoxemia subsequently contributed to substantial cardiomyocyte edema and myofilament degradation [34]. According to our available data, endotoxemia-induced heart damage was ameliorated in Vit C-pretreatment, as seen by decreased myonecrosis and edema as well as minimal inflammation. Some fields showed some muscle fibers with peripheral slightly rarified nuclei were seen in numerous fields, whereas others revealed well-organized muscle fibers and central vesicular nuclei.

According to our TEM findings, the myofibrils of endotoxemic rats’ cardiomyocytes were likewise damaged. Furthermore, certain myocytes showed striation loss in the form of indistinct Z-lines; the A band and H zone were practically invisible, and the intercalated discs’ distinctive shape was lost. Myofibril disintegration could result from the activation of calcium-induced proteinase in necrotic muscle [35]. Mitochondria are needed for various physiological activities, including the maintenance of pro- and antioxidant mechanisms that lead to the development of a wide range of cardiovascular disorders [36]. Moreover, mitochondria are responsible for creating energy, directing cellular metabolism and apoptosis through the control of oxygen and nutrients [37] With the decrease in the cytoplasmic fraction of cytochrome C, as well as a reduction in mitochondrial cytochrome C translocation, mitochondrial alterations are generated. Increased mitochondrial nitrotyrosine synthesis also was employed to indicate NO-related cardiomyocyte injury [38]. NO, in contrast, has been linked to cytotoxicity since it has been shown to disrupt mitochondrial respiratory chain enzymes and trigger mitochondrial-induced apoptosis. Variations in mitochondrial enzyme activity have been attributed to mitochondrial size changes and cristae per mitochondrion [39]. In the endotoxemic group, our findings revealed that the mitochondria’s inner membrane enlarged, leading to cristae fragmentation. In contrast, Vit C administration to the endotoxemic group revealed that the dense matrix and complete cristae among most mitochondria were maintained. However, a minority were engorged, with an irregular structure and size and were partially damaged. 

Our present research revealed that the nuclei of endotoxemic cardiomyocytes showed variable degrees of loss of chromatin density and karyolysis. Invaginations of the nuclear membrane and chromatin clumping were also observed. Cardiomyocyte disruption and their mitochondrial components contributed to perinuclear edema. This is confirmed by the evidence documented that heart ischemia causes chromatin clumping and contracted nuclei; this gives rise to cardiomyocyte necrosis [40]. The endotoxic group had malformed endothelial cells, which make up the vascular lumen. Our findings revealed capillary damage in the heart’s vasculature that could explain myofibrillar disintegration, in accordance with earlier research [41]. 

In this study, Vit C protected against endotoxin-induced heart injury by improving the cardiac ultrastructures such as the cardiomyocytes, ICDs, endothelial cells and mitochondria. This might be due to the suppression of oxidative stress cytokine production, which is in line with prior research showing that higher oxidative stress is linked to increased cytokine production [42]. 

Furthermore, our findings revealed a significant correlation between damage to the heart’s architecture, oxidative stress biomarkers (SOD and MDA), and proinflammatory cytokines (IL-6 & TNF-α), implying a correlation between the pathophysiology of endotoxemia-induced cardiac injury and these parameters and confirming that Vit C can protect against LPS-induced cardiac damage in rats. This study showed a link between endotoxin, oxidative stress, inflammatory biomarkers and cardiac injury where the antioxidant (Vit C) alters the course of cardiomyopathy in an animal model. A possible beneficial effect of antioxidants might present a new addition to the range of secondary preventive measures used in LPS-induced cardiac damage. As LPS does not explicitly act on the cardiac trusses, we intended to extend our research to evaluate the effect of Vit C against LPS-induced lung, liver, and kidney damage.

### Study Limitation

Despite these findings, there are many limitations in this research. Our data is still observational, which is essential. Furthermore, based on these findings, it is currently unclear to pinpoint the upstream mechanism by which pretreatment of endotoxin-treated rats with Vit C safeguards them from LPS-induced cardiac damage. However, more research utilizing a dose–response curve is strongly advised. Identifying alternative pathways that regulate inflammation, measurements of cardiac function and heart failure markers could also be a promising area for future research. Lastly, this study focused solely on pretreatment for Vit C’s ability to protect against LPS-induced cardiac damage. However, examining this effect on heart function and all assessed markers at different time intervals could be more informative. Additionally, this study showed only the potential protective effect of Vit C on LPS-induced cardiac damage. However, no one will anticipate endotoxemia and start taking Vit C. Vit C’s ability to protect against LPS-induced damage to other organs such as the liver, lungs, and kidneys is not studied in this research and would be included in further studies.

## 5. Conclusions

According to our findings, the administration of Vit C to endotoxin-treated rats safeguards them from LPS-induced cardiac damage by lowering the lipid peroxidation and increasing antioxidant enzymes, reducing the production of oxidative stress and inflammatory biomarkers that damage the heart architecture.

## Data Availability

The data that support this study’s findings are accessible upon request.
